# Bioactive Potential of *Cydonia oblonga* and *Diospyros kaki* Vinegars: Phenolic Content, Antioxidant and GST Inhibitory Activity, and Molecular Docking Studies

**DOI:** 10.1155/bmri/6699595

**Published:** 2025-12-02

**Authors:** Zuhal Sahin, Fatih Sonmez, Davut Avci, Tulay Duran, Nourhane A. Darwich

**Affiliations:** ^1^ Pamukova Vocational School, Sakarya University of Applied Sciences, Pamukova, Sakarya, Turkey; ^2^ Department of Physics, Faculty of Science, Sakarya University, Serdivan, Sakarya, Turkey, sakarya.edu.tr; ^3^ Department of Biological Sciences, Faculty of Science, Beirut Arab University, Beirut, Lebanon, bau.edu.lb

**Keywords:** *Cydonia oblonga*, *Diospyros kaki*, GST inhibition, molecular docking, vinegar

## Abstract

Fruits and their peels are rich sources of bioactive compounds with significant health benefits. Vinegars produced from fruits and their peels are gaining increasing interest as a functional food due to their antioxidant, antimicrobial, and potential therapeutic properties. In this study, the four vinegars (*Cydonia oblonga* pulp vinegar [COV], *Cydonia oblonga* peel vinegar [COPV], *Diospyros kaki* pulp vinegar [DKV], and *Diospyros kaki* peel vinegar [DKPV]) were produced via natural fermentation. Total phenolic content (TPC), total flavonoid content (TFC), antioxidant properties (DPPH and ABTS activity), and glutathione S‐transferase (GST) inhibitory activity of these produced vinegars were evaluated. Additionally, in order to compare the properties of these products with natural home‐style/traditional, the same content and activities of *Cydonia oblonga* fruit vinegar (COFV) and *Diospyros kaki* fruit vinegar (DKFV) were examined. The results indicated that COPV exhibited the highest TPC (842.57 ± 56.79 mg GAE/L) and TFC (248.45 ± 55.08 mg catechin/L), while DKPV showed the highest GST inhibitory activity (81.25%). All samples demonstrated significant DPPH and ABTS radical scavenging potential. Vinegars derived from peels (COPV and DKPV) generally contain higher levels of bioactive compounds than the others. Additionally, docking results indicated that the carbonyl and hydroxyl groups and aromatic rings of main components in extracts interacted extensively with GST active cite via *π*‐donor, carbon and/or conventional H‐bonds, *π*‐anion, *π*‐alkyl, *π*‐*π* T‐shaped, and amide‐*π* stacked interactions. The findings suggest that peel vinegars are a promising source of natural antioxidants and potential GST inhibitors. These findings also highlight their potential value as functional food products.

## 1. Introduction

Fruits and vegetables are an important part of the daily diet and are consumed in large quantities. Consumers value not only the nutritional value and safety of fruits but also their natural, organic, or healthy qualities [1, 2]. Fruits are rich in bioactive compounds ([3–5]. Due to the rich phenolic content of fruits, they can prevent many diseases, such as anti‐inflammatory, cancer, antihypertensive, and antiviral properties [6]. This widespread consumption of fruits also generates waste. These wastes include pulp, peel, roots, and seeds. These wastes are an abundant and affordable source of phenolic compounds [7].

Vinegar is a natural food product obtained by firstly alcoholic fermentation and then acetic acid fermentation [8]. The phenolic compounds found in vinegar originate from the raw material. This content can vary depending on the vinegar processing method and material [9]. Vinegar can be classified as grain vinegar, fruit vinegar, and alcoholic vinegar, depending on the raw material used [10]. Fruit vinegar is produced as a way of utilizing fruit by‐products in the food industry. It has been determined that the vinegars obtained in this way exhibit high‐quality taste, aroma, and bioactive properties [11]. Glutathione S‐transferases (GSTs, EC 2.5.1.18) are a class of enzymes that play an important role in detoxification by conjugating reactive electrophilic molecules with glutathione (GSH) within the cell. GSTs have cytosolic forms in most vertebrates, and these cytosolic GSTs are divided into several classes; common ones are alpha (GSTA), mu (GSTM), pi (GSTP), theta (GSTT), zeta (GSTZ), omega, and sigma [12, 13]. Studies with GSTs isolated from equine liver show different specific activity values for some substrates compared to human GSTs. For example, for general substrates such as 1‐chloro‐2,4‐dinitrobenzene (CDNB), activities are similar or sometimes even higher in equine liver [14]. All vinegars have strong antibacterial and antioxidant activity. Vinegars derived from fruits strengthen the reduced GSH antioxidant system. Vinegars protect liver cells from damage. Numerous studies have shown that vinegar is used in the treatment of certain diseases, such as hypertension, diabetes, obesity, hypercholesterolemia, antitumor properties, blood glucose control, hyperglycemia, and cancer [15–19].

In this study, the total phenolic content (TPC), total flavonoid content (TFC), antioxidant properties, and GST inhibitory activity of vinegars produced from the pulp and peel of *Cydonia oblonga* (quince) and *Diospyros kaki* (persimmon) (*Cydonia oblonga* pulp vinegar [COV], *Cydonia oblonga* peel vinegar [COPV], *Diospyros kaki* pulp vinegar [DKV], and *Diospyros kaki* peel vinegar [DKPV]) via natural fermentation were investigated. Furthermore, the molecular docking assay was also performed. All results of vinegars obtained from quince and persimmon were compared with a natural home‐style/traditional *Cydonia oblonga* fruit vinegar (COFV) and *Diospyros kaki* fruit vinegar (DKFV). While the literature generally focuses on vinegars derived from fruit flesh, this study expands existing knowledge by systematically comparing the biological activities of vinegars derived from peels. Specifically, by examining phenolic/flavonoid content, antioxidant capacity, and GST inhibition, it provides a comprehensive assessment at both the chemical and biological levels.

## 2. Materials and Methods

### 2.1. Materials

*Cydonia oblonga* (CO) and *Diospyros kaki* (DK) collected from the Pamukova district in Sakarya (40^°^ 30^′^ 30^″^ *N*, 30^°^ 9^′^ 53^″^ *E*) were used. For comparative analysis, natural home‐style/traditional COFV and DKFV were purchased from the local market. All chemicals used in the analysis were supplied by Sigma‐Aldrich (Sternheim, Germany) and Merck (Darmstadt, Germany).

### 2.2. Vinegar Production

Vinegar production was carried out using natural fermentation, a modification of the method described in the literature [11, 20]. Quince, quince peel, persimmon, and persimmon peel were used in the production. Vinegar production consists of two stages: The first stage is alcoholic fermentation, and the second stage is acetic acid fermentation. Alcoholic fermentation: 50 g of fresh fruit or peel, sterile water, and *Saccharomyces cerevisiae* (~0.25 *g*/*L*) (commercial instant yeast, product code: 51085) were mixed. It was initially adjusted to 13°Brix. It was fermented at 20 ± 2^°^ *C* under anaerobic conditions for 6–8 weeks. At the end of this stage, the alcohol content reached approximately 7–8*%*. Acetic acid fermentation: The fermented mixture was filtered. Five percent (v/v) mother vinegar was added to the filtrate. The lids are closed with a gas‐permeable system. They are incubated for 10–12 weeks under aerobic conditions at 22–25^°^ *C*. Acetic fermentation was completed when the alcohol content dropped to levels ≤ 0.5%. Vinegars were produced in three replicates from each sample under the same conditions.

### 2.3. Total Phenolic Content

The TPC was determined following the procedure described by Sonmez & Sahin [21], using the Folin–Ciocalteu colorimetric method. Briefly, 100 *μ*L of each sample (COV, COPV, COFV, DKV, DKPV, and DKFV) was mixed with diluted Folin–Ciocalteu reagent. After an initial reaction time of 3 min, 20% (w/v) aqueous sodium carbonate (Na_2_CO_3_) solution was added to each mixture. The samples were then incubated for 60 min at room temperature in the dark. Following incubation, absorbance was measured at 765 nm using a UV–Vis spectrophotometer. A standard calibration curve was prepared using gallic acid. The gallic acid concentration is in the range of 25–1000 *μ*g/mL. The gallic acid calibration chart equation was calculated as *y* = 0.0016*x* + 0.0119 (*R* ^2^ = 0.9919). Results were expressed as mg gallic acid equivalents (GAE)/L of sample.

### 2.4. Total Flavonoid Content

TFC of vinegar samples was determined according to Bakir et al. [22]. The 500 *μ*L vinegar samples taken into the tube were diluted with 2.5 mL of distilled water. Five percent NaNO_2_ solvent was added and incubated for 6 min. After incubation, 10% AlCl_3_·6H_2_O solution was added and 1 mL of NaOH (1 M) was added. The total volume was adjusted to 5 mL with distilled water. The absorbance values of the samples were determined at a wavelength of 510 nm. A catechin standard curve (7.8–1000 *μ*g/mL) was used.

### 2.5. DPPH Assay

**Figure 1:**
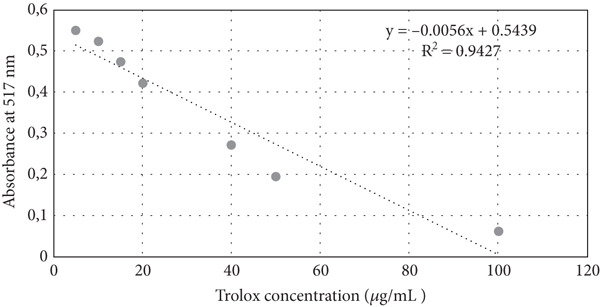
Calibration chart of Trolox equivalents used for DPPH.

The antioxidant activity of the vinegars was evaluated according to their ability to scavenge 2,2‐diphenyl‐1‐picrylhydrazyl (DPPH) free radicals using the method described by Ozdemir [23], with minor modifications. Three milliliters of DPPH methanol solution (0.06 mM) was added to 100 *μ*L of COV, COPV, COFV, DKV, DKPV, and DKFV. The mixture was vortexed and incubated in the dark at room temperature for 30 min to allow the reaction between antioxidants and DPPH radicals to occur. After incubation, the absorbance of the samples was measured at 517 nm using a UV–Vis spectrophotometer (UVmini‐1240, UV visible spectrophotometer, Shimadzu, Japan, 1 cm path length). To express antioxidant capacity in Trolox equivalents, a standard calibration curve was generated using Trolox at concentrations of 5–100 *μ*g/mL. The absorbance inhibition (%) of Trolox standards was plotted to form a linear regression equation (*y* = −0.0056*x* + 0.5439) (Figure 1). The antioxidant capacity of the samples was then calculated from the Trolox standard curve and given as *μ*mol Trolox equivalent (TE)/mL. % Inhibition values were calculated from the absorbance values obtained using the formula:

%I=1100−A sampleA control×

### 2.6. ABTS Assay

**Figure 2:**
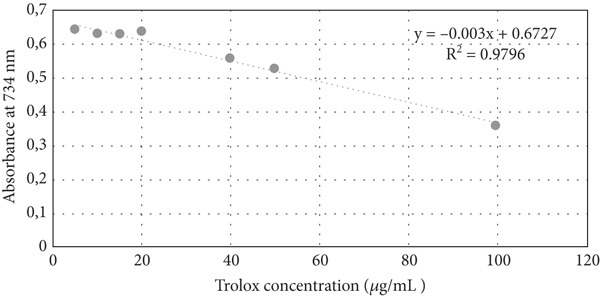
Calibration chart of Trolox equivalents used for ABTS.

The antioxidant activity of vinegars reacted with the 2,2 ^′^‐azino‐bis (3‐ethylbenzothiazoline‐6 sulfonic acid) radical was determined using the method applied by Melkis and Jakubczyk [24]. ABTS radical cation solution was prepared by incubating a mixture of ABTS and K_2_S_2_O_8_ at room temperature in the dark for 24 h. The absorbance of the resulting solution was adjusted to the desired value at 734 nm by dilution. The radical solution was added to vinegar samples. The decrease in absorbance was monitored at 734 nm for 6 min (UVmini‐1240, UV visible spectrophotometer, Shimadzu, Japan, 1 cm path length). Trolox standards were used to generate the calibration curve (Figure 2). The results were reported as *μ*mol Trolox equivalent (TE)/mL. % Inhibition values were calculated from the absorbance values obtained using the formula:

%I=1100−A sampleA control×

### 2.7. Glutathione‐S‐Transferase Inhibition

The GST inhibition assay was carried out in a cuvette containing a reaction mixture composed of 100 *μ*L of glutathione (20 mM), 150 *μ*L of CDNB (25 mM), 2.65 mL of potassium phosphate buffer (100 mM, pH 6.5), and 50 *μ*L of GST enzyme (from equine liver). Subsequently, 50 *μ*L of vinegar sample was added to the mixture. The absorbance was recorded at 340 nm initially (0 min) and at 1‐min intervals [25]. The final concentrations of the substances used in the cuvette were determined as GSH (0.67 mM), CDNB (1.25 mM), and GST (0.104 U/mL), respectively. In the GST inhibition experiment, the study was carried out at room temperature with a UV–visible spectrophotometer with a path length of 1 cm. The extinction coefficient of the reaction product at 340 nm was *ε* = 9.6 *m* *M* − 1 *c* *m* − 1. All reagents, except the vinegar sample as a control, were mixed under the same conditions. The blank was made with a mixture without enzymes and vinegar. The 100% enzyme inhibition value was determined by determining the absorbance difference. Based on the absorbance measurements, the % glutathione‐S‐transferase inhibition values were calculated as follows:

%I=ΔAbssample ΔAbscotrol×100

### 2.8. Docking Procedure

Molecular docking analyses were conducted using AutoDock4.2 [26] through the AutoDockTools (ADT 1.5.7) [27] interface to investigate the interactions between the protein and the main component chlorogenic acid for COV [9], 5‐*O*‐caffeoylquinic acid for COPV [28], gallic acid for DKV [29], and kaempferol 3‐*O*‐*β*‐rutinoside for DKPV [30]. The crystal structure of the target protein, GST (PDB ID: 7BIB), was obtained from the Protein Data Bank. Molecular docking was performed against human GSTA1 due to the availability of a high‐resolution structure, while in vitro assays utilized equine liver GST. This species/isoform difference is acknowledged as a limitation, and the docking results are therefore considered hypothesis‐generating rather than definitive. However, as all experimental assays were conducted under identical conditions with the same enzyme source, this does not affect the validity of the comparative vinegar findings. The PDB files of the compounds DKV, DKPV, COV, and COPV were generated. AutoDockTools (ADT 1.5.7) was utilized to assign Kollman charges, determine torsional flexibility by defining rotatable bonds, and combine non‐polar hydrogens with their corresponding carbon atoms. The Lamarckian genetic algorithm (LGA) [31] was employed to carry out docking calculations involving the flexible conformations of the compounds and the target protein. AutoDock 4.2 was utilized to generate the PDBQT‐formatted grid and docking parameter files for the target and ligand (target.pdbqt and ligand.pdbqt), while AutoGrid 4 was employed to define the docking site. Grid box dimensions were configured to enclose the binding site and facilitate optimal conformational mobility of the ligand. Binding interactions were analyzed by generating a 3D affinity grid at the compound site with a grid spacing of 1.0 Å. The receptor atom types considered were A, C, HD, N, OA, and SA, whereas the ligand was modeled with A, C, HD, and OA. The docking procedure employed a population size of 150, a mutation rate of 0.02, elitism of 1, a crossover rate of 0.8, a local search rate of 0.06, and up to 2,500,000 energy evaluations. Docked conformations were considered final when they satisfied a root mean square deviation (RMSD) criterion of 2 Å. The LGA and the empirical free energy scoring function provided reproducible docking results for ligands with approximately 10 flexible bonds.

DKV: The constructed grid box consisted of 80, 62, and 66 points along the *x*, *y*, and *z* dimensions, respectively, with its center located at coordinates 22.989, − 0.116, and 10.778.

DKPV: The constructed grid box consisted of 72, 64, and 66 points along the *x*, *y*, and *z* dimensions, respectively, with its center located at coordinates 22.989, − 0.116, and 10.778.

COV: The constructed grid box consisted of 72, 60, and 62 points along the *x*, *y*, and *z* dimensions, respectively, with its center located at coordinates 22.989, − 0.116, and 10.778.

COPV: The constructed grid box consisted of 80, 60, and 68 points along the *x*, *y*, and *z* dimensions, respectively, with its center located at coordinates 22.989, − 0.116, and 10.778.

The docking outcomes were analyzed to evaluate the relative stability and binding affinities of the main component. Molecular interactions between the compounds and protein residues were visualized in both 2D and 3D using Discovery Studio 4.0 [32].

### 2.9. Statistical Analysis

Statistical analyses were conducted using Minitab Statistical Software version 19.0 (State College, Pennsylvania, United States). A one‐way analysis of variance (ANOVA) followed by Tukey’s post hoc test was applied to assess statistical differences among groups. Differences were considered statistically significant at a confidence level of *p* < 0.05.

## 3. Results and Discussion

### 3.1. Total Phenolic Content and Total Flavonoid Content

The TPC and TFC of vinegar vary depending on the raw material used. Many studies show that the total phenolic content and total flavonoid content of quince/persimmon peel are higher than that of quince/persimmon pulp ([33–37]. TPC, TFC, DPPH, and ABTS antioxidant activity values of the studied vinegars are given in Table 1. The vinegars obtained from the peels have significantly higher phenolic content and flavonoid content as expected. The TPC of the vinegars were determined to be between 271.93 ± 9.62 mg GAE/L and 842.56 ± 56.79 mg GAE/L. On the other hand, TFC was determined to be between 30.31 ± 2.32 mg catechin/L and 248.45 ± 55.08 mg catechin/L. Vinegar obtained from quince peel has the highest phenolic content (842.56 ± 56.79 mg GAE/L) and flavonoid content (248.45 ± 55.08 mg catechin/L). The TPC and TFC of DKPV (669.92 ± 54.12 mg GAE/L and 52.50 ± 7.40 mg catechin/L, respectively) were determined to be significantly higher than DKV (315.75 ± 40.41 mg GAE/L and 32.46 ± 9.22 mg catechin/L, respectively) and DKFV (271.93 ± 9.62 mg GAE/L and 30.59 ± 1.68 mg catechin/L, respectively). Statistically, total phenolic content and flavonoid content show significant differences (*p* < 0.05).

**Table 1:** Total phenolic content (TPC), total flavonoid content (TFC), and antioxidant activity (DPPH, ABTS) of *Cydonia oblonga* vinegar (COV), *Cydonia oblonga* peel vinegar (COPV), *Cydonia oblonga* fruit vinegar (COFV), *Diospyros kaki* vinegar (DKV), *Diospyros kaki* peel vinegar (DKPV), and *Diospyros kaki* fruit vinegar (DKFV).

**Sample**	**TPC (mg GAE/L)**	**TFC (mg catechin/L)**	**DPPH activity**		**ABTS activity**	
			**(*μ*mol TE/mL)**	**Inhibition (%)**	**(*μ*mol TE/mL)**	**Inhibition (%)**
*COV*	398.53 ± 70.28c	30.31 ± 2.32b	20.96 ± 0.06a	69.13 ± 4.76	44.81 ± 2.40a	81.15 ± 0.44
*COPV*	842.56 ± 56.79a	248.45 ± 55.08a	21.97 ± 0.34a	73.47 ± 3.37	50.14 ± 2.80a	89.22 ± 3.40
*COFV*	373.18 ± 13.70c	65.95 ± 2.58b	12.47 ± 5.52b	21.73 ± 6.57	38.58 ± 11.52a	71.52 ± 4.95
*DKV*	315.75 ± 40.41c	32.46 ± 9.22b	21.40 ± 0.20a	70.86 ± 0.25	48.68 ± 5.67a	85.77 ± 2.59
*DKPV*	669.92 ± 54.12b	52.50 ± 7.40b	20.13 ± 1.49a	60.00 ± 6.36	52.95 ± 1.22a	94.43 ± 0.20
*DKFV*	271.93 ± 9.62c	30.59 ± 1.68b	22.53 ± 0.1a	82.91 ± 1.00	51.01 ± 3.06a	90.25 ± 3.05

*Note:* The results are presented as mean ± SD (standard deviation), with a sample size of *n* = 3. Different lowercase letters in the same column indicate significant differences (*p* < 0.05).

### 3.2. DPPH and ABTS Activities

The ABTS method is based on the transfer of hydrogen atoms and electrons, while the DPPH method is based on the reduction of DPPH in the presence of a hydrogen‐donating antioxidant [38]. While DPPH activity demonstrated statistically significant differences (*p* < 0.05), no significant difference was observed in the ABTS activity study (*p* > 0.05). While COFV (12.47 ± 5.52 *μ*mol TE/mL) demonstrated the lowest antioxidant activity, the others indicated similar effects. Graphical representations of DPPH and ABTS activity % inhibition values for investigated vinegars are given in Table 1. The antioxidant activity inhibition % values of these vinegars ranged from 21.73 ± 6.57% to 82.91 ± 1.00% for DPPH, while ranged from 82.91 ± 1.00% to 94.43 ± 0.20% for ABTS. It was observed that the % inhibition values obtained and the calculated *μ*mol TE/mL values were similar. Stoenescu and Stanica reported the DPPH activity values of commercial vinegar (36.98%) and home‐style/traditional vinegar (10.62%) [39]. All vinegars in this study demonstrated higher inhibition than home‐style/traditional vinegar, while COFV demonstrated lower inhibition than commercial vinegar. Jansone et al. reported that Japanese quince and black currant vinegars exhibited antioxidant activity of 16.63 ± 0.02 mg TE 100 mL^−1^ and 16.38 ± 0.04 mg TE 100 mL^−1^, respectively [40]. DPPH activities of the vinegars obtained in this study were determined to be lower than reported on the study by Jansone et al. ABTS activities of several berry vinegars were determined to be between 1.52 mM TE/L and 0.44 mM TE/L by Padureanu et al. [41]. ABTS activities of studied vinegars were determined to be quite high compared to several berry vinegars. The antioxidant activity results of the vinegar samples appear to be consistent with their phenolic and flavonoid contents.

### 3.3. Glutathione‐S‐Transferase Inhibition

**Figure 3:**
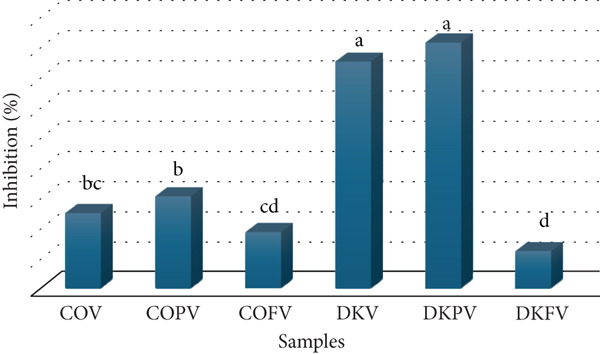
Graphical representation of GST enzyme inhibition % values for COV, COPV, COFV, DKV, DKPV, and DKFV. Lowercase letters in the chart indicate the difference between columns (*p* < 0.05).

GSTs are important enzymes involved in the detoxification process [42]. Overactivity of these enzymes is seen in various types of cancer and inhibiting them has therapeutic benefits [43]. Graphical representation of GST enzyme inhibition (%) values for investigated vinegars is given in Figure 3. GST enzyme % inhibition values for COV, COPV, COFV, DKV, DKPV, and DKFV were 25%, 30.56%, 18.75%, 75%, 81.25%, and 12.5%, respectively. Statistical differences were determined in GST enzyme inhibition values among vinegars (*p* < 0.05). Significant activity reductions were observed under test conditions. While the pH value of the buffer solution used under test conditions was 6.5, the pH values of the vinegar samples varied between 2.8 and 3.4. The final pH value under working conditions was determined to be approximately 6.2–6.5. DKPV (81.25%) shows the highest enzyme inhibition, followed by DKV (75%). For comparative analysis, home‐style/traditional quince (COFV) and persimmon vinegar (DKFV) purchased from the local market had the lowest inhibition value (18.75% for COFV, 12.5% for DKFV). Literature searches have not found any direct studies demonstrating that vinegar inhibits GST enzyme activity. Some studies and findings may be indirectly related to vinegar and its ingredients. Perfluorooctane sulfonate (PFOS) and wood vinegar (WV) were used in the treatment of planaria by Wang et al. [44]. The protective effects of PFOS on oxidative stress in planarians and WV on lipid peroxidation, antioxidant enzyme activity, and mRNA expression were investigated. It was determined that low concentrations of WV had protective effects on oxidative damage caused by PFC in planarians via changes in glutathione peroxidase (GPx), GST, and glutathione reductase (GR) activities [44]. The effects of vinegar on oxidative stress in rats fed high cholesterol were investigated by Seydim et al. [45]. Significant differences were determined in GSH‐Px levels in both TGV and TAV groups fed with vinegar [45].

### 3.4. Molecular Docking

Molecular docking studies are a powerful assay for determining the interaction potential of plant‐derived compounds and with biological targets (such as enzymes, receptors, or DNA structures) and for understanding inhibition mechanisms [26]. Bioactive compounds and the products derived from plant extracts can inhibit or reduce the function of an enzyme by binding to its active site. Docking results, such as binding sites, binding energy and affinity, and interaction types and strength, help elucidate inhibition mechanisms [46].

According to molecular docking results, protein‐ligand interactions, distance values and docking scores (binding energies) are given in Table 2. Moreover, 2D and 3D representations of the molecular interactions between protein residues and selected compounds are shown in Figure 4. The binding energies of gallic acid, kaempferol 3‐*O*‐*β*‐rutinoside, chlorogenic acid and 5‐O‐caffeoylquinic acid were observed as − 2.83, − 1.98, and − 3.38, and − 3.97 kcal/mol, respectively. Aromatic hydroxyl groups of phenolic content in extracts interacted with LYS, MET, THR, TYR and PHE amino acid residues of GST active cite via conventional H‐bond and/or *π*‐donor H‐bond interactions. In addition, the *π*‐donor H‐bonds, electrostatic and/or hydrophobic interactions such as *π*‐anion, amide‐*π* stacked, *π*‐alkyl, and *π*‐*π* T‐shaped were observed between aromatic rings (phenyl and kaempferol) and GLU, GLY, LEU, LYS, MET, and TYR amino acid residues. Furthermore, carbonyl fragments and hydroxyl groups of rutinoside and caffeoyl moieties of the main component in extracts interacted with ASN, ASP, ILE, LEU, LYS, and TYR residues of enzymes via conventional H‐bond and/or carbon H‐bond interactions.

**Table 2:** Protein–ligand interactions and distance values for the main components in DKV, DKPV, COV, and COPV.

**Substrate**	**Receptor**	**Interaction**	**Distance (Å)**	**Docking scores (binding energy, kcal/mol)**
DKV (Gallic acid)				−2.83
C=O (O)	TYR95 (OH)	Conventional H‐bond	2.57	
Ph‐O	LYS64 (O)	Conventional H‐bond	2.78	
Ph‐O	TYR49 (O)	Conventional H‐bond	3.22	
Ph‐O	LYS64 (O)	Conventional H‐bond	3.23	
Phenyl	MET51	Hydrophobic (pi‐alkyl)	4.61	
DKPV (kaempferol 3‐O‐*β*‐rutinoside)				−1.98
C=O (O)	LEU153 (O)	Conventional H‐bond	3.04	
Ph‐O	THR193 (N)	Conventional H‐bond	3.19	
Ph‐O	THR193 (OG1)	Conventional H‐bond	2.91	
Rutinoside (O)	LEU153 (O)	Conventional H‐bond	2.99	
Rutinoside (O)	ASN80 (O)	Conventional H‐bond	3.18	
Rutinoside (O)	LYS152 (CE)	Carbon H‐bond	3.69	
Rutinoside (O)	LYS152 (CE)	Carbon H‐bond	3.41	
Kaempferol	LYS152 (OH)	Pi‐donor H‐bond	3.75	
Kaempferol	LYS152 (OH)	Pi‐donor H‐bond	3.71	
Kaempferol	TYR147	Hydrophobic (pi‐pi T‐shaped)	5.09	
Kaempferol	LEU153	Hydrophobic (pi‐alkyl)	5.17	
Kaempferol	LEU153	Hydrophobic (pi‐alkyl)	5.27	
Kaempferol	LEU191	Hydrophobic (pi‐alkyl)	4.11	
COV (chlorogenic acid)				−3.38
Ph‐OH	PHE30 (O)	Conventional H‐bond	3.20	
Ph‐OH	PHE30 (O)	Conventional H‐bond	2.64	
Phenyl	GLU32 (N)	Pi‐donor H‐bond	3.63	
COPV (5‐*O*‐caffeoylquinic acid)				−3.97
C=O (O)	LYS4 (N)	Conventional H‐bond	2.75	
Ph‐OH	LYS64 (NZ)	Conventional H‐bond	3.19	
Ph‐OH	LYS64 (NZ)	Conventional H‐bond	2.99	
Caffeoyl‐OH	LYS78 (NZ)	Conventional H‐bond	2.87	
Ph‐OH	MET63 (O)	Conventional H‐bond	2.62	
Caffeoyl‐OH	ASP61 (OD1)	Conventional H‐bond	3.12	
Caffeoyl‐OH	ASP61 (OD1)	Conventional H‐bond	3.18	
*O*‐CO (O)	ILE60 (CA)	Carbon H‐bond	3.27	
Caffeoyl‐C	ASP61 (OD1)	Carbon H‐bond	3.08	
Phenyl	GLU59 (OE2)	Electrostatic (Pi‐anion)	4.35	
Phenyl	GLY62 (C,O)	Hydrophobic (amide‐pi stacked)	3.95	

Figure 42D and 3D representations of the molecular interactions between protein residues and the main components in (a) DKV, (b) DKPV, (c) COV, and (d) COPV.

**Figure:**
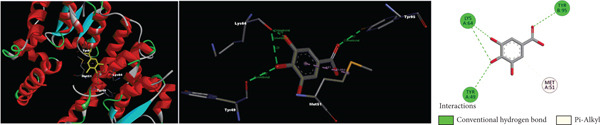
(a)

**Figure:**
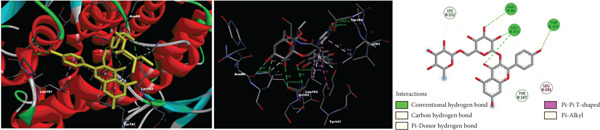
(b)

**Figure:**
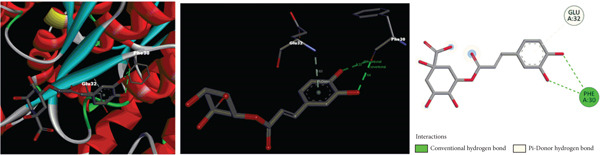
(c)

**Figure:**
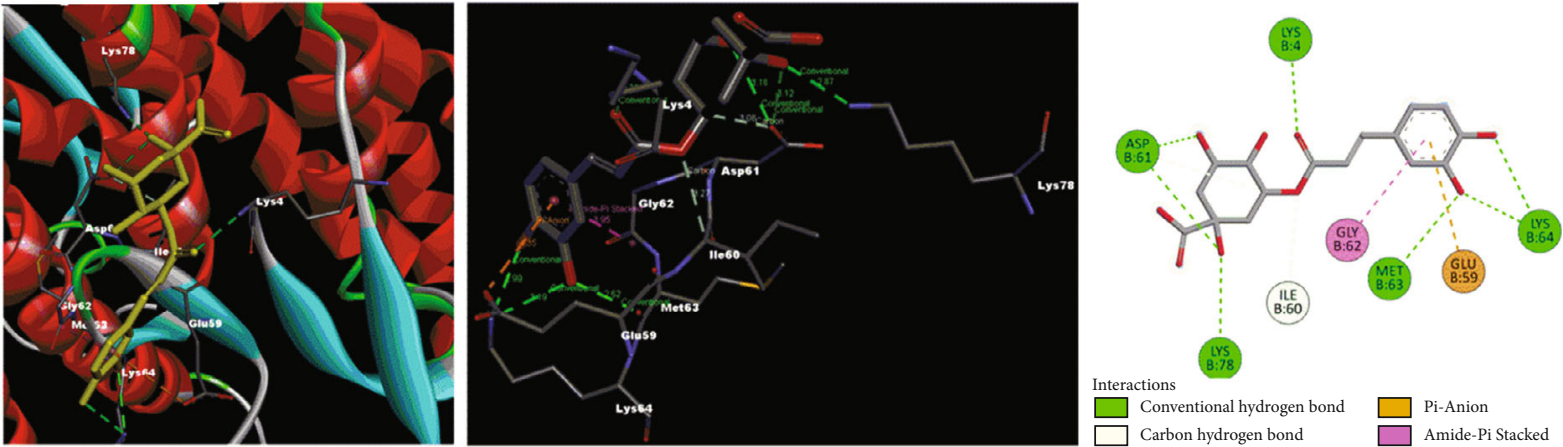
(d)

This study extends the existing literature by shifting attention from fruit‐pulp‐based vinegars to peel‐derived vinegars, demonstrating their higher levels of phenolics, flavonoids, and antioxidant capacity. By integrating GST inhibitory activity with molecular docking analyses, it not only highlights the therapeutic potential of these products but also provides mechanistic insight into enzyme–ligand interactions. Thus, the findings advance current knowledge on fruit vinegars by emphasizing the functional value of peel‐derived products and their promise as natural sources of bioactive compounds.

## 4. Conclusions

The current study demonstrates that vinegars produced from quince and persimmon peels have significantly higher total phenolic content and flavonoid content compared to pulp or commercially sourced vinegars. This improved phytochemical profile was associated with significantly strong antioxidant activity. In particular, persimmon peel vinegar exhibited the greatest glutathione S‐transferase inhibitory effect, demonstrating its potential as a functional food with bioactive properties related to oxidative stress regulation and detoxification processes. The results highlight the potential for utilizing peels in vinegar production to enhance nutritional and therapeutic value while contributing to waste reduction in the food industry. The positive effects of vinegars derived from peels on health and the elucidation of GST enzyme inhibition mechanisms can be strengthened with in vivo and clinical studies.

## Conflicts of Interest

The authors declare no conflicts of interest.

## Author Contributions

Z.S.: methodology, formal analysis, writing. F.S.: methodology, writing—review and editing. D.A.: methodology, formal analysis, software. T.D.: formal analysis. N.A.D.: writing—review and editing, and also served as the corresponding author, responsible for submission, communication with the journal, and handling revisions.

## Funding

No funding was received for this manuscript.

## Data Availability

The data that support the findings of this study are available from the corresponding author upon reasonable request.
